# T210. PSYCHOSOCIAL CORRELATES OF INTERPERSONAL PLEASURE IN SCHIZOPHRENIA-SPECTRUM PATIENTS

**DOI:** 10.1093/schbul/sby016.486

**Published:** 2018-04-01

**Authors:** Diane Gooding, Yael Ratner, Nina Mendyk, Herman Farkash, Michael Ermiyev, Michael S Ritsner

**Affiliations:** 1 University of Wisconsin-Madison; 2 Shaar Menashe Mental Health Center; 3 National Insurance Institute, Shaar Menashe Mental Health Center

## Abstract

**Background:**

Although many people with schizophrenia-spectrum disorders report high levels of social anhedonia, it is not clear what differentiates those patients who self-report social anhedonia from those who do not. Moreover, the extent to which the hedonic functioning of severely disordered patients is associated with their clinical symptoms or with personality-related factors remains unresolved.

**Methods:**

We administered the Anticipatory and Consummatory Interpersonal Pleasure Scale (ACIPS; Gooding & Pflum, 2014), a self-report measure designed to assess hedonic capacity for social and interpersonal pleasure, to 125 consecutively admitted inpatients with schizophrenia-spectrum disorder. The (81 schizophrenia, 44 schizoaffective disordered) patients were assessed in terms of their illness and symptom severity. They were also administered measures of self-efficacy (GSES; Jerusalem & Schwarzer, 1992), quality of life (Q-LES-Q-18; Ritsner et al., 2005), and recovery level (RAS-20; Salzer, 2010). Based on total ACIPS scores, two cut-off points were defined in order to classify participants as ‘normally hedonic’, ‘hypohedonic’ or ‘anhedonic’.

**Results:**

The ACIPS negatively correlated with 8 PANSS items: conceptual disorganization (P2, r=-0.24, p<0.01), hallucinatory behavior (P3, r=-0.28, p<0.01), suspiciousness (P6, r=-0.31, p<0.001), emotional withdrawal (N2, r=-0.24, p<0.01), stereotyped thinking (N7, r=-0.19, p<0.05), tension (G4, r=-0.23, p<0.01), G5 mannerism and posturing (G5, r=-0.22, p<0.05), and disturbance of volition (G13, r=-0.26, p<0.01).In addition, the ACIPS positively correlated with self-efficacy, self-esteem, perceived social support, subjective quality of life, and recovery scale scores.

**Discussion:**

The ACIPS is a reliable and valid means to measure social anhedonia in a clinical sample. The findings revealed that the self-reported hedonic functioning of schizophrenia-spectrum patients is associated with both clinical symptomatology as well as some personality-related variables. Suggestions for further clinical and research applications using the ACIPS will be provided.

